# The key role for local base order in the generation of multiple forms of China HIV-1 B'/C intersubtype recombinants

**DOI:** 10.1186/1471-2148-5-53

**Published:** 2005-10-07

**Authors:** Chi-Yu Zhang, Ji-Fu Wei, Shao-Heng He

**Affiliations:** 1 Department of Biochemistry and Molecular Biology, Jiangsu University School of Medical Technology, Zhenjiang, Jiangsu 212001, China; 2 Allergy and Inflammation Research Institute, the Medical College of Shantou University, Shantou, Guangdong, 515031, China

## Abstract

**Background:**

HIV-1 is a retrovirus with high rate of recombination. Increasing experimental studies *in vitro *indicated that local hairpin structure of RNA was associated with recombination by favoring RT pausing and promoting strand transfer. A method to estimate the potential to form stem-loop structure by calculating the folding of randomized sequence difference (FORS-D) has been used to investigate the relationship between secondary structure and evolutionary pressure in some genome. It showed that gene regions under strong positive "Darwinian" selection were associated with positive FORS-D values. In the present study, the sequences of HIV-1 subtypes B' and C, both of which represent the parent strains of CRF07_BC, CRF08_BC and China URFs, were selected to investigate the relationship between natural recombination and secondary structure by calculating the FORS-D values.

**Results:**

The apparent higher negative FORS-D value region appeared in the *gag*-*pol *gene region (nucleotide 0–3000) of HIV-1 subtypes B' and C. Thirteen (86.7 %) of 15 mosaic fragments and 17 (81 %) of 21 recombination breakpoints occurred in this higher negative FORS-D region. This strongly suggested that natural recombination did not occur randomly throughout the HIV genome, and that there might be preferred (or hot) regions or sites for recombination. The FORS-D analysis of breakpoints showed that most breakpoints of recombinants were located in regions with higher negative FORS-D values (P = 0.0053), and appeared to have a higher negative average FORS-D value than the whole genome (P = 0.0007). The regression analysis also indicated that FORS-D values correlated negatively with breakpoint overlap.

**Conclusion:**

High negative FORS-D values represent high, base order determined stem-loop potentials and influence mainly the formation of stem-loop structures. Therefore, the present results suggested for the first time that occurrence of natural recombination was associated with high base order-determined stem-loop potential, and that local base order might play a key role in the initiation of natural recombination by favoring the formation of stable stem-loop structures.

## Background

The human immunodeficiency virus type 1 (HIV-1) is a complex retrovirus, which encodes the enzyme reverse transcriptase (RT), and exhibits high mutation rates due to the lack of the DNA proofreading activity of the viral RT. HIV-1 genome is diploid, containing two plus-strand viral RNA copies that can be identical. In the process of viral DNA synthesis, template switching occurs by translocation of RT between two genomic RNAs, and results in both intra-molecular and inter-molecular recombination. If dual infections or superinfection with different strains or subtypes of HIV-1 occurs, two different RNA templates might be co-packaged into one virion, yielding a heterozygous virion. In a subsequent infection cycle, RT may switch from one template (the donor) to the other (the acceptor), producing a mosaic HIV-1 genome [1,2]. HIV-1 has high potential to form recombination variants [3,4]. The high rate of recombination is due to the frequent template switching of RT. At least 2.8 template switching events occur per genome per replication cycle was estimated previously [5]. Genetic recombination and point mutation are both important strategies to increase viral diversity, which allow HIV-1 to escape immune attack and to develop possibly drug-resistant variants [6].

Retroviral recombination generally occurs during minus-strand DNA synthesis [7]. The "Dock and Lock" model had been proposed to shed light on the mechanism of retroviral recombination. This model suggested that RT switches templates when it encounters palindrome (hairpin) structures that can induce RT to pause. RT pausing during synthesis can enhance strand transfer [1,2,8]. RNA secondary structures play an important role in the function of an RNA molecule, such as RNA-protein interactions, transcription, translation, and so on. Previous studies *in vitro *have indicated that specific RNA secondary structures were associated with strand transfer by favoring RT pausing [9,10]. However, it remains uncertain whether RNA secondary structure is involved in the generation of circulating HIV-1 recombinants.

Currently, some HIV-1 recombination variants have been identified worldwide [6]. Sixteen prevalent inter-subtype recombinants were recognized as circulating recombinant forms (CRFs) from 01 to 16, respectively [11]. Three CRFs, CRF01_AE, CRF07_BC and CRF08_BC were found in China. Of them, CRF07_BC and CRF08_BC possibly arose in Yunnan Province, and had circulated widely among injecting drug users (IDUs) [12-16]. In addition, the unique recombinant forms (URFs), between subtypes B' (Thailand variant of subtype B) and C, are epidemic among IDUs in Dehong Prefecture in western Yunnan, suggesting on-going generation of new HIV-1 intersubtype recombinants [14,15]. Most HIV-1 infected IDUs in China were unemployed, and never received any antiretroviral therapy due to lack of income [16]. Therefore, there is no drug selective pressure associated with generation of recombinants in China, and these recombinants represent the occurrence of natural recombination.

The stem-loop structure is the most important secondary structure of RNA. A method to estimate the potential to form stem-loop structure by calculating FORS-D has been used to investigate the relationship between secondary structure and evolutionary pressure [17,18]. Previous studies by Forsdyke showed that gene regions under strong positive "Darwinian" selection were associated with positive FORS-D values, reflecting the conflict between stem-loop structure potential and specific protein function [17,19-21]. In addition, our previous work found that the FORS-D values correlated negatively with *ccr5 *gene deletions, indicating that stem-loop structure influences the deletion [22]. These suggested that stem-loop structures might play an important role in mutation strategies and gene evolution. Therefore, in the present study, we selected China CRFs and URFs as a means to investigate the relationship between the secondary structure and natural recombination by analyzing the FORS-D values of HIV-1 genome.

## Results and discussion

### The distribution of FORS-D values in HIV subtype B' and C genomes

Previous studies have analyzed the local secondary structural information of some HIV-1 strains by calculating the "statistically significant" stem-loop potential, and found that different regions of HIV-1 genome had different potential to form stem-loop structures [23,24]. The regions with high stem-loop potential were generally associated with the interaction between local secondary structures and corresponding protein factors. For example, trans-acting responsive element (TAR) and Rev-responsive element (RRE), both of which are recognized by the Tat protein and Rev protein respectively, have more stable local secondary structures than other regions of HIV-1 genome [23-25]. A negative correlation between "statistically significant" stem-loop potential and sequence variability (substitutions) was observed in the HIV-1 genome. In the regions with higher negative FORS-D values, indicating that base order favors stem-loop potential, the rate of base substitutions tend to be lower. Contrarily, higher positive FORS-D values decrease stem-loop potential and is functionally important, because the rate of base substitutions increases [20,23-25].

Genetic recombination is another important pathway to generate variability for HIV-1. Previous studies found that local stem-loop structure enhanced the occurrence of template switching of RT [2,10,26]. To assess whether local stem-loop structure is involved in the generation of natural HIV-1 recombination, FORS-D analysis was applied to estimate the potential of HIV-1 sequences to form stem-loop structures. The FORS-D value represents a base order-determined stem-loop potential, and provides a measure of the contribution of base order alone to the formation of stem-loop structure [18]. The FONS value determines the trend of total stem-loop potential.

Yunnan Province of China has a high HIV-1 prevalence among IDUs and generates multiple forms of HIV-1 intersubtype recombinants [14,16]. Because most HIV-1 infected IDUs are unemployed, they almost never receive any antiretroviral therapy [16]. Therefore, the recombinant forms circulated among IDUs indicate the occurrence of the natural recombination between HIV-1 subtypes B' and C without drug selective pressure. To investigate the relationship between local stem-loop potential and natural recombination, two closely related HIV-1 strains, 95IN21068 and RL42, which are known to be parent strains of China inter-subtype B' and C recombinants, were selected as objectives to analyze FORS-D.

**Figure 1:**
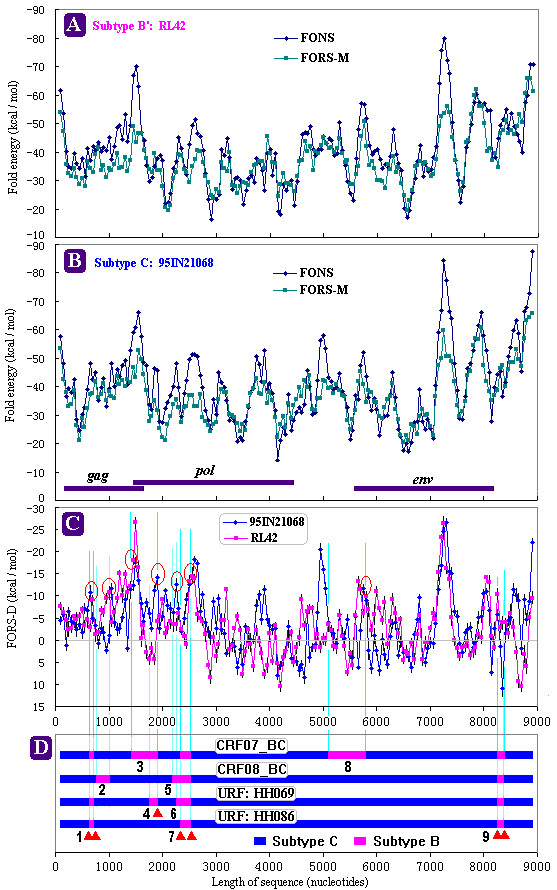
FORS-D analysis of HIV-1 subtypes B' and C. **A**, FONS and FORS-M values of HIV-1 subtypes B'; **B**, FONS and FORS-M values of HIV-1 subtypes C; **C**, FORS-D values (± SE) of both HIV-1 subtypes B' and C; **D**, the location of all breakpoints and mosaic pattern of four full-length HIV-1 recombinants circulated in China. FONS, FORS-M and FORS-D values were calculated in successive 200 nt windows, each of which overlapped the previous window by 150 nt. Vertical dashed lines indicated the location of breakpoints in FORS-D distributions. The red open circles in FORS-D distributions (**C**) pointed out these recombination breakpoints located in regions with higher negative FORS-D values. The solid triangle indicated the breakpoints shared by more than one HIV-1 recombinant form (**D**). The inserted recombined fragments were numbered from 1 to 9 (**D**).

FONS and FORS-M values found in RL42 and 95IN21068 are shown in part **A **and **B **of Figure 1, respectively. Both HIV-1 strains appeared to have similar trends in FONS and FORS-M values. FORS-M values were relatively constant (For subtype B': median value was -35. 79 kcal/mol with a range from -65.78 to -19.6 kcal/mol; for subtype C: median value was -36.42 kcal/mol, with a range from -65.6 to -20.25 kcal/mol). However, a large fluctuation was observed with FONS values. Several higher negative FONS values appeared in 5' and 3' termini, 3' end of the *gag *gene, and around nucleotide 7250 in the *env *gene. These results were consistent with the previous observations in other HIV-1 strains [20,23-25]. Figure 1C shows FORS-D values for both 95IN21068 and RL42. Except for a few regions, HIV-1 subtype B' and C genomes appeared to have similar distribution of FORS-D values. The windows with higher negative FONS values accordingly appeared to have higher negative FORS-D values. The fluctuations of FONS values observed in the whole genome of the both HIV-1 subtypes were largely base-order dependent, as reflected in the FORS-D values (Fig. 1A, B and 1C ). The highest negative FORS-D values of both sequences occurred around nucleotide 7250 in RRE region (nucleotide 7081–7432).

For each HIV-1 subtype, the distribution of FORS-D values differed in different regions of the gene. The apparent higher negative FORS-D value region occurred in the *gag *gene and at the 5' end of the *pol *gene (nucleotide 0–3000) (For subtype B': *gag*-*pol *region: -5.403 ± 0.8155 kcal/mol; whole genome: -3.165 ± 0.4886 kcal/mol, P = 0.0217. For subtype C: *gag*-*pol *region: -6.495 ± 0.6398 kcal/mol; whole genome: -3.775 ± 0.5035 kcal/mol, P = 0.0045). However, an intense fluctuation of positive and negative FORS-D values around the abscissa was observed in the region from the 3' part of *pol *gene to *env *gene (nucleotide 3000–8000). This region encodes RT, integrase, envelope glycoproteins gp120 and gp41, other important regulatory (Tat and Rev) and accessory (Vpr, Vif, Vpu, and Nef) proteins. They determine HIV-1 replication and efficient infection, and are exposed immediately to the human immune system and under strong positive "Darwinian" selection [27]. Previous studies on retroviral genes [19], MHC genes [20], snake venom phospholipase A_2 _[21] and other genes [17], had showed that a region under strong positive selection exhibited generally positive FORS-D values. Our results supported the observation that FORS-D value was associated with evolutionary pressure [17,19-21].

### The relationship between the HIV-1 B'/C intersubtype recombination and stem-loop potential

**Figure 2:**
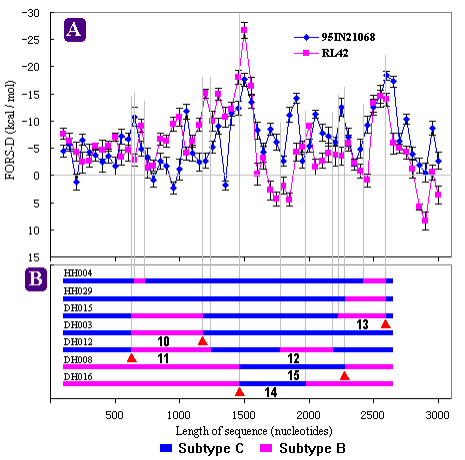
The location of all breakpoints of seven 2600-nt URFs circulated in China in FORS-D distributions of HIV-1 subtypes B' and C. **A**, FORS-D values (± SE) of both HIV-1 subtypes B' and C; **B**, the location of all breakpoints. FORS-D values were calculated in successive 200 nt windows, each of which overlapped the previous window by 150 nt. The positions of recombination were shown as boxes in **B**. Vertical dashed lines indicated the location of breakpoints in FORS-D distributions. The solid triangle indicated the breakpoints shared by more than one HIV-1 recombinant form (**B**). The numbers of inserted recombined fragments were continued from 10 to 15 (**B**). The fragment numbers occurred in Fig. 1D were not shown.

*In vitro *studies using the HIV-1 derived vector system indicated that HIV-1 genome has high rate of recombination and hot spots for recombination occurrence [3,5]. The hot spots were located in stable hairpin structures [4]. However, the previous observation did not indicate whether the occurrence of HIV-1 CRFs and URFs in worldwide distribution correlates with secondary structures of RNA templates due to sequence difference between vector and circulated strains. To assess whether stem-loop structures are involved in occurrence of natural recombination, we selected subtype B' RL42 and subtype C 95IN21068, both of which represent the parent strains of existing CRFs and URFs in China, to carry out FORS-D analysis. Currently, besides CRF07 and 08, only two other full-length sequences of URFs circulated in China are available. These four existing recombinants, representing four different recombination variants, were selected and analyzed using the Simplot software. Their mosaic patterns are shown in Fig. 1D. The breakpoints of recombination are identified in the FORS-D distribution of RL42 and 95IN21068 (Fig. 1C and 1D) by fine vertical dashed lines. In addition to these four full-length sequences, other URFs were also analyzed despite the availability of only *gag*-RT region (about 2600 nucleotides) [14]. Figure 2 shows their partial mosaic map and FORS-D distribution in breakpoints.

In total, 15 different inserted recombined fragments were identified in China HIV-1 B'/C intersubtype recombinants (Fig. 1D and 2B) [12-15]. Thirteen (86.7 %) of these mosaic fragments occurred in the higher negative FORS-D value region (nucleotide 0–3000) of parent genomes, where the *gag *gene and 5' end of the *pol *gene are located. On the other hand, because several shared breakpoints were observed in these mosaic molecules, which were confirmed by our previous reports [14,15], 15 mosaic fragments only contained 21 unique breakpoints. For example, fragment 5, 6, 7 and 13 shared the 3' end breakpoint. Among these breakpoints, 17 (81 %) also located in this higher negative FORS-D value region. This strongly indicates that natural recombination did not occur randomly throughout the HIV genome, and that there might be preferred (or hot) regions or sites for recombination [3,5]. These observations suggest an association between recombination and high negative FORS-D values (Fig. 1C and Fig. 2A).

In order to further confirm the relationship between recombination and high negative FORS-D values, FORS-D values of corresponding breakpoints of parent sequences were calculated as described in the Methods. The FORS-D values of breakpoints of 15 fragments were shown in Table 1. For most fragments, at least one breakpoint of each fragment was found to be located in regions with higher negative FORS-D values. Two exceptions were fragment 11 and 12. They had at least one breakpoint located in the regions of higher negative FORS-D values in one parent subtype, and at least one breakpoint located in the regions of negative FORS-D values (close to the mean of whole genome) in another parent subtype (Table 1). Twenty-one breakpoints appeared to more favor occurring in higher negative FORS-D values region (69 %, P = 0.0053), and negative FORS-D values region (92.9 %, P = 0.0007). In addition, the average FORS-D values of breakpoints (-6.29 ± 0.81 kcal/mol) also appeared to be more negative than whole genome (-3.47 ± 0.35 kcal/mol) (P = 0.0079), suggesting that the values of breakpoints were significantly different from that of whole genomes. The data indicated that recombination preferentially occurred in high negative FORS-D regions.

**Table 1:** The FORS-D values of breakpoints of inserted recombined fragments occurred in China HIV-1 B'/C intersubtype recombinants.

**No. fragment**^#^	**Recombinant strains**	Subtype B'* (kcal/mol)		Subtype C* (kcal/mol)	
		
		**Left (5'-)**	**Right (3'-)**	**Left (5'-)**	**Right (3'-)**
**1**	CRF07, HH069, HH086	3.04	-8.12	-7.07	-8.63
**2**	CRF08	-1.52	-6.38	-7.00	-9.20
**3**	CRF07	-19.88	-10.43	-14.15	-15.27
**4**	HH069	3.48	-10.43	-3.76	-15.27
**5**	CRF08, HH029	-3.97	-13.29	-5.09	-18.91
**6**	HH069	-7.15	-13.29	-9.62	-18.91
**7**	CRF07, HH086	-1.07	-13.29	-0.37	-18.91
**8**	CRF07	-0.92	-5.56	-1.32	-8.10
**9**	CRF07, CRF08, HH069, HH086	-9.94	-5.19	-5.97	-6.02
**10**	DH003, DH015	-0.90	-9.30	-2.98	-3.88
**11**	DH012	-0.90	-10.70	-2.98	-3.67
**12**	DH012	4.13	-2.93	-5.32	-7.27
**13**	DH015	-7.15	-13.29	-9.62	-18.91
**14**	DH016	-19.88	-10.43	-14.15	-15.27
**15**	DH008	-19.88	-3.97	-14.15	-5.09

# The fragment numbers were consistent with those of Fig. 1D and 2**B**. * The FORS-D values of breakpoints were calculated as described in the Methods. The average FORS-D values of subtype B' and C HIV-1 genome were -3.17 and -3.78, respectively. The value of < -3.17 (subtype B') or < -3.78 (subtype C) represents the region with higher negative FORS-D value.

*In vitro *experiments indicated that strand transfer of RT involved RT pausing and triggering retroviral recombination [1,26]. Further evidence showed that secondary structures of RNA template, especially, hairpin or stem-loop structures play a key role in RT pausing and strand transfer [2,10,26]. Two obligatory strand transfers of RT had been observed to occur in terminal sequences of viral genome with stable hairpin structures and high stem-loop potential [9]. The hairpin structure facilitates RT pausing, which stimulates RT-RNase H activity and results in donor template degradation. Pause-induced donor template degradation initiates strand transfer. Then, strand transfer is thought to progress through a two-step mechanism, first acceptor invasion, then primer terminus transfer. Two models, the kissing hairpin interaction model and the "Dock and Lock" model, have been proposed to explain this mechanism [1,26]. Both models emphasize the key role of hairpin structure in strand transfers. High negative FORS-D value, representing high base order-determined stem-loop potential, occurred generally in one or both acceptor and donor sites (Fig. 1C, 2A, and Table 1), which was supported by previous *in vitro *observation [2].

**Figure 3:**
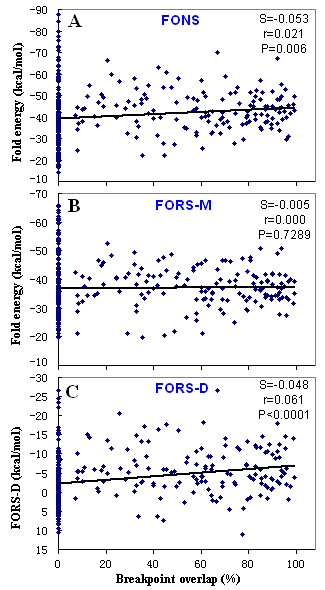
Linear regression analysis of the FONS (**A**), FORS-M (**B**) and FORS-D (**C**) value against the degree of the breakpoint overlap (%). Data (FONS, FORS-M and FORS-D) were from the breakpoints of four full-length, HIV-1 representative recombinant forms circulated in China. Parameters of the least-squares line shown in each figure were slope (**s**), the correlation coefficient (**r**), and the probability (P value) that the slope of the line is not significantly different from zero (***P***).

Further evidence for the location of breakpoints was provided by plotting FONS, FORS-M, and FORS-D values of each window against its percentage of breakpoint overlap. Figure 3 shows the linear regression analysis of the relationships between FONS (**A**), FORS-M (**B**), and FORS-D (**C**) values (kcal/mol) and breakpoint overlap. In form Fig. 3, we observed that plots for FORS-M were horizontal(r = 0.0003), and plots for both FONS and FORS-D slopes were slightly diagonal (r = 0.053 and 0.061, respectively). The slopes of the least-squares regression lines for FONS and FORS-D values were significantly greater than zero (P = 0.006 for FONS and P < 0.0001 for FORS-D). The regression analysis indicated that FONS and FORS-D values correlated negatively with breakpoint overlap, and that the correlation coefficient, as well as P value, also supported these relationships (Fig. 3). FONS value represents the total stem-loop potential, and FORS-D provides a measure of the contribution of base order alone to the stem-loop potential of a sequence. Negative FORS-D values imply that local base order favors the formation of stable stem-loop structures. Therefore, the observation above suggested for the first time that occurrence of natural recombination was associated with high base order-determined stem-loop potential, and local base order was likely to be important for the initiation of natural recombination by favoring the formation of stable hairpin structures [1]. In addition, the present results supported the previous observation that hairpin structures were involved in retroviral recombination [2,10,26]. However, a further study to extend the FORS-D analysis to other intersubtype recombinants (CRFs and URFs) circulated in other countries and regions of the world should be conducted.

### Does the information of local base order expressed by FORS-D values play a key role in the selection of evolutionary strategies for reducing the selective pressure?

Two CRFs and multiple forms of URFs have been detected in Yunnan Province of China, where needle or syringe sharing among IDUs is popular [14,16]. Needle or syringe sharing increase the risk of dual virus infections and subsequent recombination between different subtypes of viruses. To date, however, little is known about whether or not these new recombinants are associated with stronger infectivity and higher replication ability. We do know that the location of natural recombination is not random throughout the HIV genome, and there are some specific hot spots for recombination situated in the genome. Previous experimental observations *in vitro *revealed that hairpin structures increase the rate of recombination between viruses [2,8,10,26]. Here, our results support that previous notion, and indicated firstly that natural recombination was associated primarily with high base order-determined stem-loop potential (FORS-D values).

FORS-D value tends to fluctuate around zero and determines the trend of FONS value. It contains a large amount of evolutionary information about nucleic acids, and generally appears to be negative number [17]. Negative FORS-D values favor the formation of stem-loop structure and are widely distributed in long genomic segments from a variety of species. Positive FORS-D values, represent the conflict of evolutionary pressures on base order, and occur more frequently in regions under positive Darwinian selection, such as promoter regions, exons and so on [17,19-21]. The regions with positive FORS-D values indicate a tendency for local base order to support protein encoding function rather than formation of stem-loop structure. Therefore, FORS-D value generally appears to correlate positively with base substitution densities and *d*_N_/*d*_S _ratio [17]. On the other hand, our previous and present studies showed that FORS-D values were associated with the occurrence of deletion [22] and recombination mutations, which suggests that local base order is involved in the occurrence of both recombination and deletion. Because positive FORS-D values correlate with high ratio of base substitution and negative FORS-D values for recombination or deletion, we can deduce that local base order plays a critical role in the selection of gene evolutionary pathways. However, further evidence is required to support this hypothesis.

## Conclusion

By analyzing the FORS-D values of HIV-1 subtypes B' and C, both of which represent the parent strains of CRF07_BC, CRF08_BC and China URFs [12-14], we found that most breakpoints of these recombinants were located in regions with higher negative FORS-D values, and appeared to have a higher negative average FORS-D value than for the whole genome. The regression analysis indicated further that FORS-D values correlated negatively with breakpoint overlap. These results suggested for the first time that occurrence of natural recombination was associated with high base order-determined stem-loop potential, and that local base order might play a key role in the initiation of natural recombination of HIV-1 by favoring the formation of stable stem-loop structures. Combining with previous reports that FORS-D correlates positively with the ratio of base substitution [17,19-21], we could deduce that local base order might play a critical role in the selection of gene or genome evolutionary pathways, and determines the evolutionary strategies adopted by gene or genome to reduce the selective pressure.

## Methods

### Sequences and recombination analysis

In this study, the sequences of RL42, 97CN001, 97CNGX-7F and URFs, were retrieved from GenBank (Table 2). RL42, 97CN001 and 97CNGX-7F are generally used as representative strains of China HIV-1 subtypes B [28], CRF07_BC [12] and CRF08_BC [13], respectively. Sequences of RL42, 97CN001, 97CNGX-7F and URFs were aligned with subtype reference sequences using Clustal X 1.8 [29]. The full-length sequences of subtype reference isolates are available in the Los Alamos database [11]. The recombination breakpoints were analyzed by using Simplot software (version 2. 5) [30]. The parameters were used as follows: Window size: 200 bps; Step size: 10 bps; Tree algorithm: Neighbor; Distance model: Kimura; Bootstrap replicate: 100; Reference type: 50 % consensus.

**Table 2:** Sequences of HIV-1 strains used in the present study.

**Subtype**	**Sequence name**	**GenBank accession number**	**References**
B'	RL42	U71182	[28]
CRF07_BC	97CN001	AF286226	[12]
CRF08_BC	97CNGX-7F	AY008716	[13]
URF	HH086	AP005207	[15]
URF	HH069	AP005206	[15]
URF	HH004	AB090998	[15]
URF	HH029	AB090999	[15]
URF	DH003	AB078705	[14]
URF	DH008	AB078707	[14]
URF	DH012	AB078710	[14]
URF	DH015	AB078712	[14]
URF	DH016	AB078713	[14]

### FORS-D analysis of HIV-1 subtypes B'and C

The previous studies had described the application of FORS-D analysis in detail [17,18]. In brief, two factors, base composition and base order, contribute to stem-loop formation of a nucleic acid molecule. So the total stem-loop potential of a sequence can be divided into the contribution of base composition alone and the contribution of base order alone to form stem-loop structure. For a natural sequence, FONS, FORS-M and FORS-D represent total stem-loop potential, base composition-determined stem-loop potential and base order-determined stem-loop potential, respectively. FONS values are calculated by using computer program, RNAstructure (version 3.6) [31], which based on free energy minimization to find a theoretical optimum secondary structure. FORS-M value is the mean minimum free energy value of the 10 randomised sequences generated from the same window. Ten randomised sequences were obtained using the shuffle program included in the on-line software package SMS [32]. FORS-D is the difference between FONS and FORS-M, and closely corresponds to "statistically significant" stem-loop potential developed by Le et al. for analyzing potential RNA folded substructures [33].

Because CRF07_BC, CRF08_BC and most URFs are B'/C inter-subtype recombinants with mostly subtype C and a few small subtype B' segments [12-14], we selected RL42 and 95IN21068 for FORS-D analysis. RL42 and 95IN21068 are related closely to those recombinants in phylogenetic evolution and generally used as a subtypes B' and C sequence reference, respectively. Both sequences lack the 5' long terminal repeats (LTRs) and are available from GenBank under accession numbers AF067155 and U71182, respectively. To analyse the FORS-D, each sequence was divided into 177 successive 200-nucleotide windows. Each overlapped the previous window by 150 nucleotides. For each 200-nucleotide window, both FONS value and FORS-M value were calculated using RNAstructure software. Then, the difference between FONS and FORS-M (FONS less FORS-M) generated FORS-D value.

### FORS-D analysis of recombination breakpoints

The position of each breakpoint was obtained using the Simplot software (version 2.5) with 200-nucleotide window size and 10-nucleotide step size. So three successive 200-nucleotide windows were selected to calculate FORS-D values of recombination breakpoints. The position of each breakpoint was used as the center of the middle window, and each window overlapped the previous one by 190 nucleotides. The average of three windows represents the FORS-D value of each recombination breakpoint.

### Statistical analysis

All statistical analysis was conducted using GraphPad Prism version 2.00 (Biomedical Sciences, Creighton University). The FORS-D location of breakpoints was analyzed by the χ^2 ^test. The differences of FORS-D values of recombination breakpoints and *gag*-*pol *gene region with whole genomes were compared by the t test.

## Abbreviations

HIV-1, human immunodeficiency virus type 1; RT, reverse transcriptase; IDUs, injecting drug users; CRFs, circulating recombinant forms; URFs, unique recombinant forms; FORS-D, folding of randomized sequence difference; FONS, folding of natural sequence; FORS-M, folding of randomized sequence mean; TAR, trans-acting responsive element; RRE, Rev-responsive element; *d*_N_/*d*_S_, nonsynonymous-to-synonymous rate ratio.

## Authors' contributions

CYZ and JFW conceived and designed the study, performed the collection and bioinformatic analysis of the data; CYZ drafted the manuscript. SHH supervised and coordinated the whole project. All authors have read and approved the final manuscript.
